# Supplementary material for: The Actigraphy-Based Identification of Premorbid Latent Liability of Schizophrenia and Bipolar Disorder
Source: Sensors (Basel). 2023 Jan 14;23(2):958. doi: 10.3390/s23020958 (PMC9863012; doi:10.3390/s23020958)
Supplement: Supplementary file 1 [file sensors-23-00958-s001.zip › Wavelet transformation.pdf]

Continuous wavelet transform was applied to the recorded activity time series, *f(t)*, to quantify the multiscale structure of the actograms. The wavelet map was defined by the correlation coefficients of the time series *f(t)* and the wavelet function $\Psi(t)$, while * stands for complex conjugate:

$$c(u,s)=\int_{-\infty}^{\infty} f(t)\frac{1}{\sqrt{s}}\Psi^{*}\left(\frac{t-u}{s}\right)dt$$
,

The calculated $c(u,s)$ is a two-dimensional function of the translation parameter *u*, scanning along the original time variable *t*, and *s* is a scale parameter (“time window”). In our analysis, the Morlet wavelet, with $\omega_c = 10$ and $\gamma_b = 2$, was applied:

$$\Psi(t) = \frac{1}{\sqrt{\pi\gamma_b}} e^{-\frac{t^2}{\gamma_b}} e^{-i\omega_c t},$$

where $\omega_c / 2\pi$ corresponds to the center frequency, and γ_b to the bandwidth of the transform. Characteristic activity amplitudes corresponding to the actual time window were calculated according to

$$P(s) = \int_u abs\left(c(s,u)\right)^2 du$$
.

P(s) was determined from concatenated sleep periods of 5 subsequent nights, and the scale variable *s* spanned from 1s to 200s in linear scale
