# Supplementary figures and images for: The Actigraphy-Based Identification of Premorbid Latent Liability of Schizophrenia and Bipolar Disorder
Source: Sensors (Basel). 2023 Jan 14;23(2):958. doi: 10.3390/s23020958 (PMC9863012; doi:10.3390/s23020958)

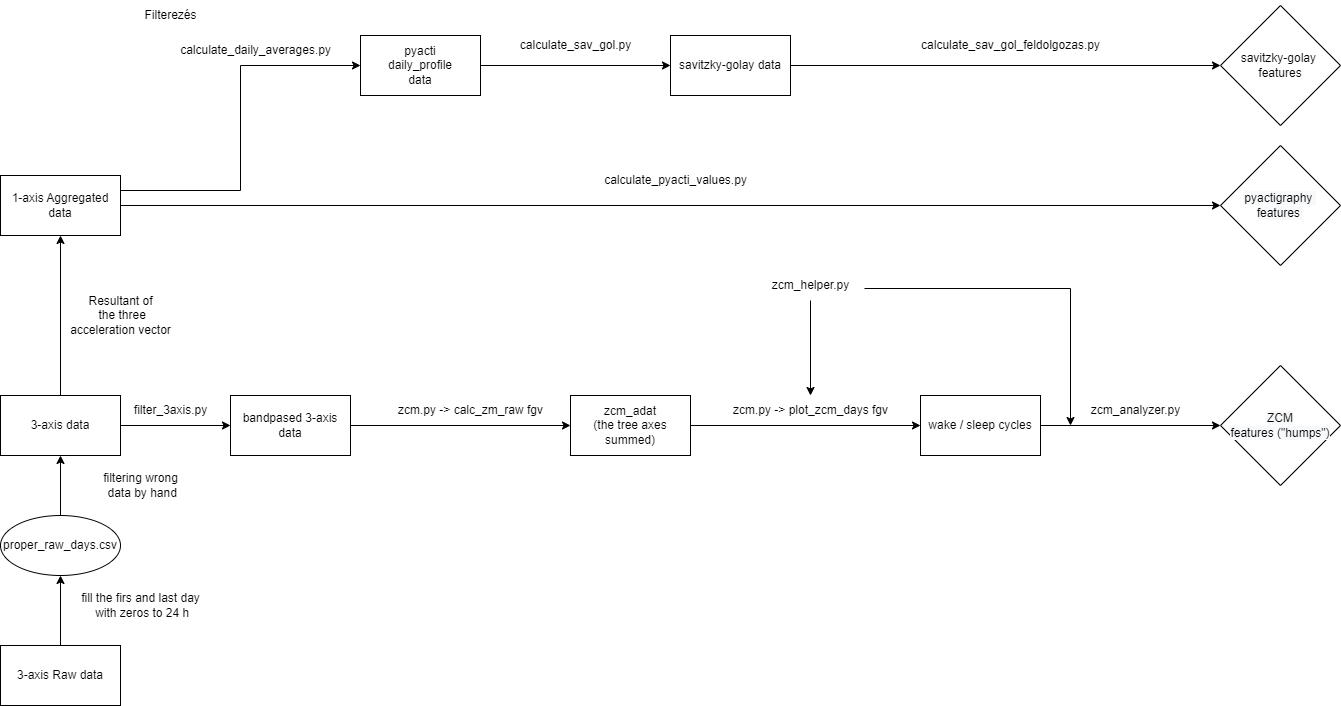

Supplement: Supplementary file 1 [file sensors-23-00958-s001.zip › aktigraf_features.png]
